# The impact of aripiprazole on neurocognitive function in individuals at clinical high risk for psychosis: A comparison with olanzapine and non-antipsychotic treatment

**DOI:** 10.1192/j.eurpsy.2025.2459

**Published:** 2025-05-22

**Authors:** JiaHui Zeng, Andrea Raballo, JiaYi Ye, YuQing Gao, WenJun Su, YanYan Wei, XiaoChen Tang, LiHua Xu, YeGang Hu, Dan Zhang, HuiRu Cui, YingYing Tang, XiaoHua Liu, HaiChun Liu, Tao Chen, ChunBo Li, JiJun Wang, TianHong Zhang

**Affiliations:** 1 Shanghai Mental Health Center, Shanghai Jiaotong University School of Medicine, Shanghai Intelligent Psychological Evaluation and Intervention Engineering Technology Research Center (20DZ2253800), Shanghai Key Laboratory of Psychotic Disorders, Shanghai, China; 2 Faculty of Biomedical Sciences, Università della Svizzera Italiana (USI), Lugano, Switzerland; 3 Department of Health and Social Care, Cantonal Socio-Psychiatric Organization (OSC), Mendrisio, Switzerland; 4 Department of Automation, Shanghai Jiao Tong University, Shanghai, China; 5 Big Data Research Lab, University of Waterloo, Waterloo, ON, Canada; 6 Labor and Worklife Program, Harvard University, Cambridge, MA, USA; 7 Center for Excellence in Brain Science and Intelligence Technology (CEBSIT), Chinese Academy of Science, Shanghai, PR China; 8 Institute of Psychology and Behavioral Science, Shanghai Jiao Tong University, Shanghai, PR China

**Keywords:** antipsychotics, aripiprazole, clinical high risk for psychosis (CHR), cognitive performance, olanzapine

## Abstract

**Background:**

Clinical high risk for psychosis (CHR) is often managed with antipsychotic medications, but their effects on neurocognitive performance and clinical outcomes remain insufficiently explored. This study investigates the association between aripiprazole and olanzapine use and cognitive and clinical outcomes in CHR individuals, compared to those receiving no antipsychotic treatment.

**Methods:**

A retrospective analysis was conducted on 127 participants from the Shanghai At Risk for Psychosis (SHARP) cohort, categorized into three groups: aripiprazole, olanzapine, and no antipsychotic treatment. Neurocognitive performance was evaluated using the MATRICS Consensus Cognitive Battery (MCCB), while clinical symptoms were assessed through the Structured Interview for Prodromal Syndromes (SIPS) at baseline, 8 weeks, and one year.

**Results:**

The non-medicated group demonstrated greater improvements in cognitive performance, clinical symptoms, and functional outcomes compared to the medicated groups. Among the antipsychotic groups, aripiprazole was associated with better visual learning outcomes than olanzapine. Improvements in neurocognition correlated significantly with clinical symptom relief and overall functional gains at follow-up assessments.

**Conclusions:**

These findings suggest potential associations between antipsychotic use and cognitive outcomes in CHR populations while recognizing that observed differences may reflect baseline illness severity rather than medication effects alone. Aripiprazole may offer specific advantages over olanzapine, underscoring the importance of individualized risk-benefit evaluations in treatment planning. Randomized controlled trials are needed to establish causality.

## Introduction

Recent studies have increasingly focused on the pre-first-episode psychosis phase, known as the clinical high risk (CHR) for psychosis [1]. Research within this domain aims to identify individuals before their initial psychotic episode and to prevent the progression to full psychosis [2]. A meta-analysis indicates that approximately 25% of CHR individuals convert to psychosis within 3 years, underscoring the need for early identification [3]. Research demonstrates that neurocognitive deficits can function as biomarkers for predicting psychosis conversion in CHR individuals [4, 5], and exhibit practical utility in the context of psychosis risk assessment [6, 7]. While some variances have been noted, our previous research has generally corroborated earlier conclusions [8] that baseline deficits in specific cognitive domains can predict later psychosis onset, with visual learning emerging as a particularly robust independent predictor of subsequent psychosis development [9]. Having established potential neurocognitive biomarkers, it is essential to consider antipsychotic (AP) treatment effects on cognition in CHR. Evidence suggests APs may negatively impact cognition and function in CHR [10, 11]. This raises the hypothesis that APs could have detrimental cognitive effects, specifically in certain domains.

The efficacy of various antipsychotic medications in enhancing neurocognitive function remains uncertain. A previous study [12] shows inconsistent medication effects on neurocognition between CHR and other disorders characterized by psychosis. This variability may be attributed to the distinct pharmacological profiles of each medication, which are likely to influence cognitive outcomes differently [13, 14]. Given inconsistent findings [8, 15–17], a critical unanswered question is how neurocognitive impairment is associated with medication status across different psychotic states. However, no studies compare the impacts of these medications on cognitive trajectories in CHR. Aripiprazole and olanzapine have distinct pharmacodynamic profiles. Aripiprazole acts as a partial D2 receptor agonist with dopamine system stabilizing properties, while olanzapine is primarily a 5-HT2A/D2 antagonist with significant affinity for H1 and M3 receptors, potentially leading to different cognitive outcomes [18]. In our previous research [19], aripiprazole was identified as the focal point due to its highest prescription rate (31%) among CHR individuals. Olanzapine, having the second-highest prescription rate (22%), provides a meaningful comparison, enabling an assessment of the differential impacts of these medications on cognitive functions. This study also incorporates data from a non-medicated group, to assess the relative effects of drug treatment versus no treatment, thereby elucidating the implications of antipsychotic use.

This study represents the first attempt to compare neurocognitive performance among CHR individuals taking aripiprazole, olanzapine, and those not on antipsychotics (AP-). Our objectives were: (1) to assess the two treatment groups across cognitive domains, symptoms, and functioning compared to AP- CHR, and (2) to investigate if cognitive changes are associated with symptom improvement. We hypothesized: (1) differing cognitive, symptomatic, and functional trajectories among groups; (2) distinct aripiprazole and olanzapine effects due to mechanisms; (3) cognitive benefits in CHR result from symptom improvement.

## Material and methods

### Participants and data collection

This observational study utilized data from the Shanghai At Risk for Psychosis (SHARP) study, including data collected from individuals identified as CHR at the Shanghai Mental Health Center since 2016 (clinicaltrials.gov ID NCT04010864) [20]. The study received approval from the research ethics committee of SMHC, with written informed consent provided by adult subjects. For participants under 18 years of age, their parents signed consent forms on their behalf, and the youths also provided informed assent. The 127 CHR participants included in the current analysis were confirmed through face-to-face interviews and fulfilled at least one of three prodromal syndrome criteria: brief intermittent psychotic syndrome, attenuated positive symptom syndrome, or genetic risk and deterioration syndrome. The eligibility requirements encompassed: (i) age below 45 years; (ii) individuals under 18 years accompanied by a parent or legal guardian; (iii) the ability to provide informed consent (or assent for those under 18 years); (iv) a minimum of six years of primary education; and (v) no prior use of psychotropic medications at the study enrollment. Exclusion criteria included: (i) severe somatic diseases (e.g., pneumonia, cancer, or heart failure); (ii) intellectual disability; or (iii) a history of substance abuse or dependence, such as methamphetamine use. Any individuals who were diagnosed with a specific psychotic disorder during the course of the study were considered to have experienced a ’conversion’ to psychosis. Consequently, they were no longer included in the ongoing investigations of the CHR cohort to ensure the analysis distinctly reflected the pre-conversion high-risk state. Further details regarding the SHARP methodology can be found in the work of Zhang et al. [21, 22].

Olanzapine and aripiprazole were selected for comparison in this study due to prior research indicating they are the most commonly prescribed antipsychotics for CHR populations [19]. Following baseline assessments and CHR diagnosis, some participants were prescribed antipsychotics by their treating psychiatrists while others remained unmedicated. To minimize confounding factors, the study included a group of AP- CHR individuals who were not receiving antipsychotic treatment to provide a comparative analysis with those receiving medication. The sample included 45 CHR individuals prescribed aripiprazole, 39 prescribed olanzapine, and 43 CHR individuals who were not on any antipsychotic treatment, assessed at baseline, 8 weeks, and 1 year.

### Measurement

The SIPS, which includes a general interview, SOPS, and Global Assessment of Functioning (GAF), was used to identify individuals with CHR. SOPS includes four symptom domains: positive, negative, disorganization, and general symptoms. In this study, we used the Chinese version of the SIPS, which has high reliability and validity [22, 23]. At one year follow-up, 13 of the remaining 127 CHR individuals had progressed to full psychosis (six were taking olanzapine, five were taking aripiprazole, and two were AP- individuals). Conversion to psychosis was defined using the Presence of Psychotic Symptoms in SIPS (POPS) [24] criteria.

Neurocognitive status was evaluated using the standardized MATRICS Consensus Cognitive Battery (MCCB) [25] at baseline (before any antipsychotic treatment), and at 8 weeks and 1 year after study entry. The MCCB tests comprised seven cognitive domains, with respective outcome measures: (1) Speed of processing (SOP; three measures were obtained: Trail Making Test, Brief Assessment of Cognition in Schizophrenia: Symbol Coding and Category Fluency); (2) Attention/Vigilance (Continuous Performance Test-Identical Pairs, CPT-IP); (3) Working memory (Wechsler Memory Scale-III: Spatial Span, WMS-III); (4) Verbal learning (Hopkins Verbal Learning Test-Revised, HVLT-R); (5) Visual learning (Brief Visuospatial Memory Test-Revised, BVMT-R); (6) Reasoning and problem-solving (Neuropsychological Assessment Battery: Maze, NAB); (7) Social cognition (Mayer-Salovey-Caruso Emotional Intelligence Test: Managing Emotions, MSCEIT). This study emphasizes raw scores because MCCB T-scores only extend to 20 (normative values generated in MCCB computerized printouts are linear estimates that may reflect cognitive development with different degrees of accuracy at different ages) [26].

### Medication and demographic information

**Figure 1.**
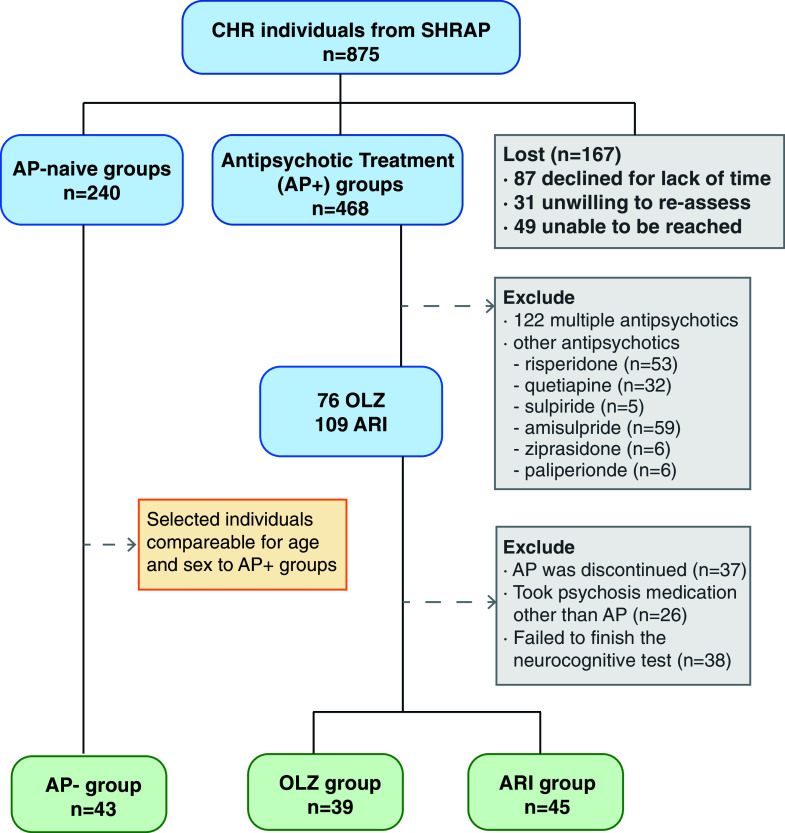
Flowchart of CHR individuals through the trial. AP-: CHR individuals not using antipsychotics. Others: CHR individuals who used other antipsychotics alone or mixed antipsychotics; OLZ: CHR individuals using Olanzapine alone; ARI: CHR individuals using Aripiprazole alone.

CHR individuals were recruited from those seeking mental health services who had not received prior antipsychotic treatment. Subsequent antipsychotic prescriptions followed the CHR evaluation, in accordance with standard clinical practice. Information regarding the use of antipsychotics and antidepressants, along with demographic details such as sex, age, and years of education, was extracted from medical records. Participants were informed that this was an observational study involving naturalistic follow-up with no intervention or remuneration. CHR participants were categorized based on their pre-existing treatments into one of three groups: olanzapine, aripiprazole, or antipsychotic-naïve (AP−). The consistency of antipsychotic use during the follow-up period was confirmed through face-to-face interviews, ensuring only those adhering to their prescribed regimen were included in the analyses. The selection process and the criteria for exclusion are detailed in Figure 1.

### Statistical analysis

Quantitative variables were expressed as mean (SD), and qualitative variables as frequencies (%). Demographic and clinical characteristics across different antipsychotic treatment groups were compared using one-way ANOVA for normally distributed variables with equal variances, confirmed by Levene’s test. For variables that did not meet these assumptions, or were not normally distributed, the Kruskal-Wallis test was applied. Post hoc analyses for ANOVA were conducted using the S–N-K method, while significant results from the Kruskal–Wallis test were further examined using Dunn’s test with Bonferroni correction. Differences between groups for categorical variables were assessed using the Chi-squared test, and independent *t*-tests were used for two-group comparisons of continuous variables.

To analyze the changes in cognitive scores from baseline to weeks 8 and year 1, we employed a one-way analysis of covariance (ANCOVA), adjusting for baseline scores and the duration of treatment. This method allowed us to control for initial differences and to assess the adjusted mean differences over time, which are reported as Least Square Mean (LSM) Changes. The LSM represents the average expected performance in each treatment group, adjusted for baseline scores and treatment duration, providing a fair comparison across groups. Post-hoc comparisons were conducted using the Student–Newman–Keuls (SNK) method whenever the ANOVA indicated significant differences between groups. This was used to identify which specific pairs of groups differed significantly in their LSM Changes.

To investigate the longitudinal effects of antipsychotic treatments on cognitive, and clinical symptoms and outcomes, we utilized linear mixed-effects models (LMMs). Model selection was guided by the Akaike Information Criterion (AIC) and the Bayesian Information Criterion (BIC), comparing various configurations of random effects and fixed interactions. The optimal model, chosen for its low AIC and BIC, effectively balanced explanatory power and simplicity. This model included fixed effects for the treatment group, time, their interaction, and daily olanzapine equivalent (OLAeq) dosage as a covariate, alongside random intercepts for each participant to address intra-individual variability.

To assess the relationships between changes in cognitive domains and clinical symptoms or functions, we first checked the normality of each variable using Shapiro-Wilk tests. Depending on the results, we used Pearson’s correlation for normally distributed data and Spearman’s rank correlation for data that did not follow a normal distribution.

## Results

### Participant characteristics

From an initial sample of 875 CHR individuals participating in the SHARP-extended study, 109 individuals were prescribed aripiprazole, and 76 were on olanzapine. After excluding individuals who did not actually use the medication, those who switched to other medications, and those who did not complete the cognitive tests, we retained 45 individuals on aripiprazole and 39 on olanzapine. We then selected 43 antipsychotic-naïve individuals (AP–) from the SHARP study, with age and sex profiles suitable for comparative analysis with the antipsychotic-medicated individuals (AP+) (Figure 1). The groups did not differ significantly in terms of age, sex, education, or antidepressant use, as demonstrated in Table 1. At baseline, the aripiprazole group received a significantly higher mean daily olanzapine equivalent (OLAeq) [27] dose than the olanzapine group. The AP– group demonstrated superior cognitive performance in Speed of Processing (Sop) and Attention & Vigilance (CPT-IP) compared to both the aripiprazole and olanzapine groups. The olanzapine group exhibited significantly more severe symptoms and a lower functional level, along with poorer scores in Verbal Learning (HVLT-R) and Reasoning and Problem Solving (NAB), compared to the AP- group, which showed the best clinical performance and functional levels.

**Table 1.** Demographic and clinical characteristics of the samples at baseline

Variable	AP- (*n* = 43)	Aripiprazole (*n* = 45)	Olanzapine (*n* = 39)	H/X^2^/F/t	*p*
^a^Age, mean (SD), y	20.98(5.93)	18.04(3.64)	19.26(5.84)	*H* = 4.68	0.096
^b^Sex, *n* (%)					
Male	23(53.5)	19(42.2)	22(56.4)	*X* ^2^ = 1.64	0.441
Female	20(46.5)	25(55.6)	17(43.6)		
^c^Education, mean (SD), y	11.88(3.42)	11.11(3.09)		10.36(3.18)	*F* = 2.28	0.107
SIPS variables at baseline					
^a^GAF score, mean(SD)	59.84(10.34) *	56.29(7.16)		54.00(7.81) *	*H* = 7.57	**0.023**
^c^Positive symptoms, median, mean(SD)	7,8.05(3.73) *	10,9.93(3.88)		10,10.10(4.10)	*F* = 3.57	**0.031**
^c^Negative symptoms, median, mean(SD)	9,9.76(6.08) *	13,12.91(5.93)		13,13.03(5.30)	*F* = 4.28	**0.016**
^c^Disorganization symptoms, median, mean(SD)	4.5,4.88(3.18) *	6,6.11(3.14)		7,6.64(3.65) *	*F* = 3.04	0.052
^c^General symptoms, median, mean (SD)	8,7.43(4.09)	9,8.85(2.59)		11,10.26(3.12) *	*F* = 10.52	**0.005**
^c^SOPSTAL, median, mean(SD)	32,30.12 (14.03) *	40,37.53(10.84)		39,40.03(11.78)	*F* = 2.28	0.107
MCCB variables at baseline					
^a^Sop, mean (SD)	57.42(9.50)*	52.69 (9.12)		50.62 (8.68)	*H* = 12.52	**0.002**
^a^CPT-IP, mean (SD)	53.49 (8.29)*	49.53 (7.97)		46.97 (10.00)	*H* = 12.89	**0.002**
^a^WMS-III SS, mean (SD)	46.79 (12.49)	45.11 (11.87)		44.18 (10.78)	*H* = 0.92	0.631
^a^HVLT-R, mean (SD)	51.19 (8.97)*	48.67 (8.23)		44.33 (9.77)*	*H* = 11.55	**0.003**
^a^BVMT-R, mean (SD)	57.14 (8.49)	55.18 (7.01)		51.38 (11.35)	*H* = 5.98	0.050
^a^NAB, mean (SD)	57.98 (8.68)	53.89 (10.46)		51.92 (9.67)*	*H* = 7.99	**0.018**
^a^MSCEIT-ME, mean (SD)	35.46 (8.05)	34.73 (5.54)		33.79 (5.87)	*H* = 0.32	0.853
^b^Converted, *n* (%)	2(4.7)	5(11.11)		6(15.38)	*X* ^2^ = 2.62	0.27
^d^Daily antipsychotic dose in OLAeq, mean (SD), mg/d	_	14.26(7.31)		10.21(6.42)	*t* = 3.89	**0.001**
^d^Use time of antipsychotics within 2 months, mean (SD), mg/week	_	7.89(0.75)		7.49(1.57)	*t* = 1.46	0.13
^d^Use time of antipsychotics within 12 months, mean (SD), mg/week	_	40.60(13.38)		36.18(16.76)	*t* = 1.32	0.19
^b^Antidepressants use, *N*%	9(20.9%)	9(20.0%)		9(23.1%)	*X* ^2^ = 0.12	0.94

*Note*: a: Kruskal–Wallis Test, Dunn’s test (Post Hoc), Bonferroni (Correction); b: Chi-square (Pearson Chis-Square); c: One-way ANOVA, S-*N*-K (Post Hoc); d: Independent Samples Test.

Abbreviations: SIPS: Structured Interview For Prodromal Syndromes; MCCB: MATRICS Consensus Cognitive Battery; Sop: Speed of Processing; CPT-IP: Attention & Vigilance; WMS-III SS: Working Memory; HVLT-R: Verbal Learning; BVMT-R: Visual Learning; NAB: Reasoning & Problem Solving; MSCEIT-ME: Social cognition; OLAeq: Olanzapine Equivalent (mg); *p < 0.05.

### Changes in clinical symptoms or outcomes and functioning in the three groups

In an ANOVA of SOPS and GAF scores for each group (Supplementary Figure 1), the aripiprazole group showed significant differences from baseline in positive symptoms, disorganization symptoms, general symptoms, total SOPS scores, and GAF scores at both 8 weeks and 1 year, with negative symptoms only showing significant changes from baseline at 1 year. For the olanzapine group, scores for positive symptoms, negative symptoms, general symptoms, and total SOPS were significantly lower at 1 year compared to baseline, while GAF scores were significantly higher. The AP– group demonstrated higher GAF scores than those at baseline at both time points.

LMMs demonstrated significant main-time effects on SOPS component scores, total SOPS, and GAF (Supplementary Table 1). Olanzapine had worse disorganization symptoms and overall symptoms, and lower GAF versus the AP– group. Significant interactions emerged between the AP– and olanzapine groups for positive symptoms, disorganization symptoms, and total SOPS.

Spearman’s correlation found GAF declines negatively associated with conversion at 8 weeks (*r* = −0.365, *p* = 0.013) and 1 year (*r* = −0.355, *p* = 0.005) for all subjects. Within the olanzapine group, GAF decline also negatively correlated with conversion at 8 weeks (*r* = −0.474, *p* = 0.005) and 1 year (*r* = −0.474, *p* = 0.026). Cognition changes did not correlate significantly with clinical outcomes.

### Neurocognitive changes in the three groups

To thoroughly assess the neurocognitive impact of antipsychotic treatments, our analysis encompassed two distinct statistical approaches. Results derived from a one-way analysis of covariance (ANCOVA) are displayed in Table 2, offering insights into the immediate cognitive effects of antipsychotic treatments by adjusting for baseline scores and treatment duration. This analysis revealed differential impacts on cognitive domains such as processing speed, attention, verbal learning, and visual learning. The AP- group demonstrated the greatest improvements in reasoning, problem-solving, and social cognition from baseline. They also had significantly greater gains than the aripiprazole group in processing speed, verbal learning, and visual learning at 8 weeks, and the olanzapine group in processing speed and attention at 8 weeks (all *p* < 0.05). Notably, the aripiprazole group improved more than olanzapine in attention at 8 weeks (*p* < 0.05).

**Table 2.** Least square mean (LSM) change from baseline on MCCB in CHR individuals at weeks 8 and year 1 of treatment with three groups^a^

	AP-						Olanzapine						Aripiprazole						
	Baseline		Week 8(*N* = 33)		Week 48(*N* = 22)		Baseline		Week 8(*N* = 32)		Week 48(*N* = 23)		Baseline		Week 8(*N* = 35)		Week 48(*N* = 22)		
Domain	Mean	SD	ΔLSM	SE	ΔLSM	SE	Mean	SD	ΔLSM	SE	ΔLSM	SE	Mean	SD	ΔLSM	SE	ΔLSM	SE	*p* ^b^
Sop	57.42	9.50	5.23	0.99	4.15	1.40	50.62	8.68	0.15	0.97	1.91	1.33	52.69	9.12	−0.52	0.92	0.35	1.34	A, B, E, F
CPT-IP	53.49	8.30	4.69	1.23	6.26	1.81	46.97	10.00	−0.69	1.24	2.54	1.76	49.53	7.97	2.68	1.10	1.64	1.64	A, B, B’, E, G
WMS-III SS	46.79	12.49	1.64	1.54	4.61	1.67	44.18	10.78	1.13	1.53	3.36	1.63	45.11	11.88	0.90	1.46	3.07	1.66	-
HVLT-R	51.19	8.97	0.97	1.38	4.36	1.43	44.33	9.78	−2.36	1.38	1.14	1.41	48.67	8.23	–4.22	1.29	3.22	1.37	A, F
BVMT-R	57.14	8.49	1.97	1.12	3.07	1.20	51.38	11.35	−0.19	1.11	–0.16	1.20	55.18	7.01	0.18	1.04	–0.68	1.20	A, F
NAB	57.98	8.68	4.01	1.33	1.96	1.46	51.92	9.67	2.36	1.33	2.68	1.40	53.89	10.46	0.58	1.29	–0.08	1.39	C
MSCEIT-ME	35.46	8.05	–0.96	1.22	3.65	1.75	33.79	5.87	–0.65	1.75	8.38	2.96	34.73	5.54	–0.21	1.43	4.49	2.69	C’

Abbreviations: ΔLSM: Least Square Mean (LSM) Change; Sop: Speed of Processing; CPT-IP: Attention & Vigilance; WMS-III SS: Working Memory; HVLT-R: Verbal Learning; BVMT-R: Visual Learning; NAB: Reasoning & Problem Solving; MSCEIT-ME: Social cognition.

a Analyzed using a general linear model. Least square mean data are raw scores, adjusting for baseline and weeks of treatment.

b Week 8: A = overall effect between all groups, *p* < 0.05; B = improvement from baseline with Non-Antipsychotic, *p* < 0.05; C = improvement from baseline with Olanzapine, *p* < 0.05; D = improvement from baseline with Aripiprazole, *p* < 0.05; E = Non-Antipsychotic versus Olanzapine, *p* < 0.05; F = Non-Antipsychotic versus Aripiprazole, *p* < 0.05; G = Olanzapine versus Aripiprazole, *p* < 0.05; Week 24: A’ = overall effect between all groups, *p* < 0.05; B’ = improvement from baseline with Non-Antipsychotic, *p* < 0.05; C’ = improvement from baseline with Olanzapine, *p* < 0.05; D’ = improvement from baseline with Aripiprazole, *p* < 0.05; E’ = Non-Antipsychotic versus Olanzapine, *p* < 0.05; F’ = Non-Antipsychotic versus Aripiprazole, *p* < 0.05; G’ = Olanzapine versus Aripiprazole, *p* < 0.05.

In order to capture the longitudinal trajectory of these effects and accommodate the complexity of repeated measures within individuals, we applied LMMs for in-depth analysis. The results, shown in Table 3, indicate that the AP- group performed significantly better than olanzapine on processing speed, attention/vigilance, verbal learning, visual learning, and reasoning/problem-solving. Moreover, aripiprazole led to greater improvements than olanzapine in visual learning. Over the course of the study, significant main time effects were found for processing speed, attention/vigilance, and social cognition from baseline to 1 year, and for reasoning from baseline to 8 weeks. The aripiprazole and olanzapine groups had significant interaction effects on verbal learning, while the AP- and olanzapine groups interacted on attention/vigilance over 8 weeks. Pairwise comparisons further revealed the AP- group scored significantly higher than aripiprazole on processing speed, verbal learning, and reasoning after Bonferroni correction (all *p* < 0.05).

**Table 3.** Linear mixed-effect model of MCCB

Domain [β(SE)/F]	Sop	CPT-IP	WMS-III SS	HVLT-R	BVMT-R	NAB	MSCEIT-ME
Ari-Olan	2.35(1.96)	2.53(2.10)	0.21(2.47)	3.36(1.99)	4.13(1.89)*	1.24(2.20)	0.33(2.25)
None-Olan	6.22(2.30)**	8.54(2.46)***	4.12(2.88)	8.89(2.32)***	5.05(2.20)*	7.58(2.58)**	4.39(2.62)
8weeks-Baseline	1.41(1.12)	0.11(1.29)	2.13(1.68)	−0.34(1.38)	1.81(1.22)	3.67(1.32)**	−0.06(1.82)
1year-Baseline	3.28(1.27)*	3.53(1.45)*	3.65(1.91)	2.95(1.56)	1.81(1.38)	2.88(1.50)	8.18(2.22)***
Ari-Olan×8weeks-Baseline	−1.68(1.55)	2.58(1.74)	−0.71(2.32)	−3.76(1.90)*	−1.32(1.68)	−2.99(1.85)	0.42(2.37)
None-Olan×8weeks-Baseline	1.96(1.57)	4.3(1.78)*	−1.37(2.35)	0.09(1.93)	−0.48(1.70)	−1.25(1.85)	−1.34(2.22)
Ari-Olan×1 year-Baseline	−2.71(1.81)	−1.55(2.02)	−1.06(2.71)	0.36(2.22)	−2.58(1.96)	−2.79(2.14)	−4.04(2.96)
None-Olan×1year-Baseline	−0.69(1.82)	1.02(2.07)	−0.51(2.72)	−0.45(2.23)	−0.05(1.97)	−0.80(2.14)	−4.95(2.65)

Abbreviations**:** β: estimated effect; SE: Standard Error; *F*: F value; Sop: Speed of Processing; CPT-IP: Attention & Vigilance; WMS-III SS: Working Memory; HVLT-R: Verbal Learning; BVMT-R: Visual Learning; NAB: Reasoning & Problem Solving; MSCEIT_ME: Social cognition; Ari-Olan: Aripiprazole group versus Olanzapine group; None-Olan: Non-antipsychotic group versus Olanzapine group.* *p* < 0.05; ** *p* < 0.01; *** *p* < 0.001.

### Relationship between three groups’ clinical symptoms or changes in functioning and neurocognitive changes

Pearson’s and Spearman’s correlations were utilized to examine relationships between neurocognitive and clinical symptom changes over 8 weeks and 1 year, adapting to each variable’s distribution. In the full sample, visual learning correlated with disorganization symptoms at 8 weeks (*r* = 0.334, *p* = 0.033). The significantly correlated items at 1 year included social cognition and positive symptoms (*r* = −0.547, *p* = 0.028), working memory and negative symptoms (*r* = −0.368, *p* = 0.008), working memory and disorganization (*r* = −0.291, *p* = 0.038), and attention/vigilance and general symptoms (*r* = 0.308, *p* = 0.037). Additionally, the GAF score was associated with working memory at 1 year (*r* = 0.319, *p* = 0.024).

In the AP– group, reasoning/problem-solving correlated with disorganization symptoms at 8 weeks (*r* = −0.547, *p* = 0.023). Significant 1-year AP- correlations were working memory and negative symptoms (*r* = −0.660, *p* = 0.019), disorganization symptoms (*r* = −0.588, *p* = 0.044), and general symptoms (*r* = −0.683, *p* = 0.014). The GAF score was associated with the speed of processing at 8 weeks (*r* = −0.466, *p* = 0.038) and verbal learning at 1 year (*r* = −0.790, *p* = 0.004).

In the aripiprazole group, correlations at 8 weeks included reasoning/problem-solving and negative symptoms (*r* = 0.626, *p* = 0.017), visual learning and disorganization symptoms (*r* = −0.757, *p* = 0.002), and verbal learning and general symptoms (*r* = −0.644, *p* = 0.013). At 1 year, reasoning/problem-solving correlated with general symptoms (*r* = 0.458, *p* = 0.048).

In the olanzapine group, at 8 weeks, attention/vigilance correlated with positive symptoms (*r* = −0.715, *p* = 0.030) and disorganization symptoms (*r* = −0.701, *p* = 0.035). The GAF score was associated with attention/vigilance at 8 weeks (*r* = −0.753, *p* = 0.019).

## Discussion

### Summary of findings

This observational study explored how antipsychotic medications affect specific neurocognitive functions in CHR individuals. While the broader impacts of antipsychotics are well-documented, comparisons of their effects on distinct neurocognitive domains within CHR populations remain limited. By examining data from two antipsychotic treatments and a non-medicated group, our study provides insights into the variable impacts of these treatments on neurocognitive outcomes. Our findings show significant improvements in clinical symptoms, neurocognitive functions, and overall performance among the non-medicated group compared to those receiving olanzapine and aripiprazole after we controlled for relevant factors. Notably, the antipsychotic group presented with more severe symptoms initially, which may indicate a greater inherent risk of progression to psychosis. Despite this association, our stance, in line with previous studies [10, 15, 28], remains that there is a need for prudence when prescribing antipsychotics to CHR individuals, mindful of the distinct impacts of treatment as compared to those in a first-episode psychosis phase. Moreover, a key innovation in our study is the notable advantage in visual learning seen with aripiprazole over olanzapine, following similar adjustments. Our analysis further suggests that changes in neurocognitive functions are closely linked to variations in clinical symptoms and functional outcomes [10, 19].

### Clinical characteristics and conversion to psychosis

Our ANOVA analysis showed that CHR individuals treated with aripiprazole and olanzapine experienced significant improvements in both clinical symptoms and functioning. In contrast, the AP– group only showed improvements in functional assessments. However, subsequent verification using LMMs indicated that the AP– group actually outperformed the olanzapine group in terms of both clinical symptoms and overall functioning. This pattern was similarly observed in the neurocognitive improvement measures. These findings may partly stem from individual differences that can obscure the underlying relationships between the variables of interest, as suggested by our correlation analysis [29]. Therefore, these results underscore the complexity of predicting treatment outcomes, and highlight that less robust statistical methods could lead to divergent and potentially misleading conclusions. Additionally, we noted that the average conversion rate was 15% in the AP+ group versus 5% in the AP− group. It is crucial to recognize that participants were not randomly assigned to these groups and that the AP+ group had more severe baseline symptoms. Consequently, these factors could influence conversion rates. Our findings suggest a possible association between antipsychotic use and increased conversion rates in CHR populations [10, 15, 30], but they also indicate that this could be due to more symptomatic individuals being more likely to receive medication.

### Changes in neurocognitive function and possible pharmacological mechanisms

This study employed both ANCOVA and LMMs to evaluate the neurocognitive impact of antipsychotic treatments. The ANCOVA analysis, adjusting for baseline scores and treatment duration, assessed the immediate cognitive effects of the treatments. In contrast, LMMs captured the longitudinal trajectory of these effects, dealing with the complexity of repeated measures within individuals. The AP− group consistently showed better performance than the antipsychotic groups, in line with previous evidence suggesting that antipsychotic treatment does not improve or may even worsen cognition in CHR individuals [8, 31, 32]. Recent research by Allott et al. [33] has highlighted that antipsychotics may induce visual, motor, and cognitive side effects through multiple pathways including dopamine blockade, anticholinergic burden, and neuroinflammation, which could significantly impact cognitive performance.

In our comparative analysis of specific antipsychotics, while aripiprazole showed superior improvements in attention over olanzapine at 8 weeks, the most notable finding was the enhancement in visual learning observed across multiple time points and random effects in LMMs. Given that CHR is a chronic condition requiring long-term intervention, treatments providing sustained benefits deserve particular emphasis. Therefore, the consistent improvements in visual learning with aripiprazole compared to olanzapine represent an especially meaningful clinical distinction.

However, while this study provides valuable insights into the specific impacts of aripiprazole and olanzapine, the broader context of antipsychotic treatment in neurocognition remains complex. Data remain limited regarding the comparative neurocognitive effects of these medications in early psychosis. According to Wang et al. [34], both aripiprazole and olanzapine can improve working memory, visual learning and memory, and processing speed in schizophrenia. One study found no differences between aripiprazole and olanzapine on cognition in psychotic disorders [35]. However, other research showed inconsistent results [12, 36, 37]. These variations may relate to differences in sample size, disease stage and course, initial medical care, and research methods.

Building on our study’s results, we further explored the potential pharmacological mechanisms underlying these cognitive effects. Although both aripiprazole and olanzapine are classified as second-generation antipsychotics, they exhibit distinct pharmacodynamic properties. Compared to aripiprazole, olanzapine has a lower affinity for D2, 5-HT1A, and 5-HT7 receptors, but a higher affinity for 5-HT2A, 5-HT2C, M3, and H1 receptors [18]. Earlier evidence indicates aripiprazole may improve psychosis and cognition by stabilizing dopamine (DA) systems. It acts as an antagonist in hyperdopaminergic brain regions (e.g. striatum) while displaying agonistic properties in hypodopaminergic areas (e.g. prefrontal cortex) [14, 38, 39]. Positron emission tomography (PET) studies show aripiprazole occupancies are very high at striatal D2 receptors but lower at 5-HT1A/5-HT2A receptors [40]. Research also supports the major role of striatal D2 receptors in rewarding cognitive initiation and visual discrimination learning [41–43]. Our findings align with evidence highlighting front striatal dopaminergic mechanisms in antipsychotic-related cognitive impacts. Further research should clarify the neurochemical bases underlying the differential effects of antipsychotics on domains like visual learning.

**Figure 2.**
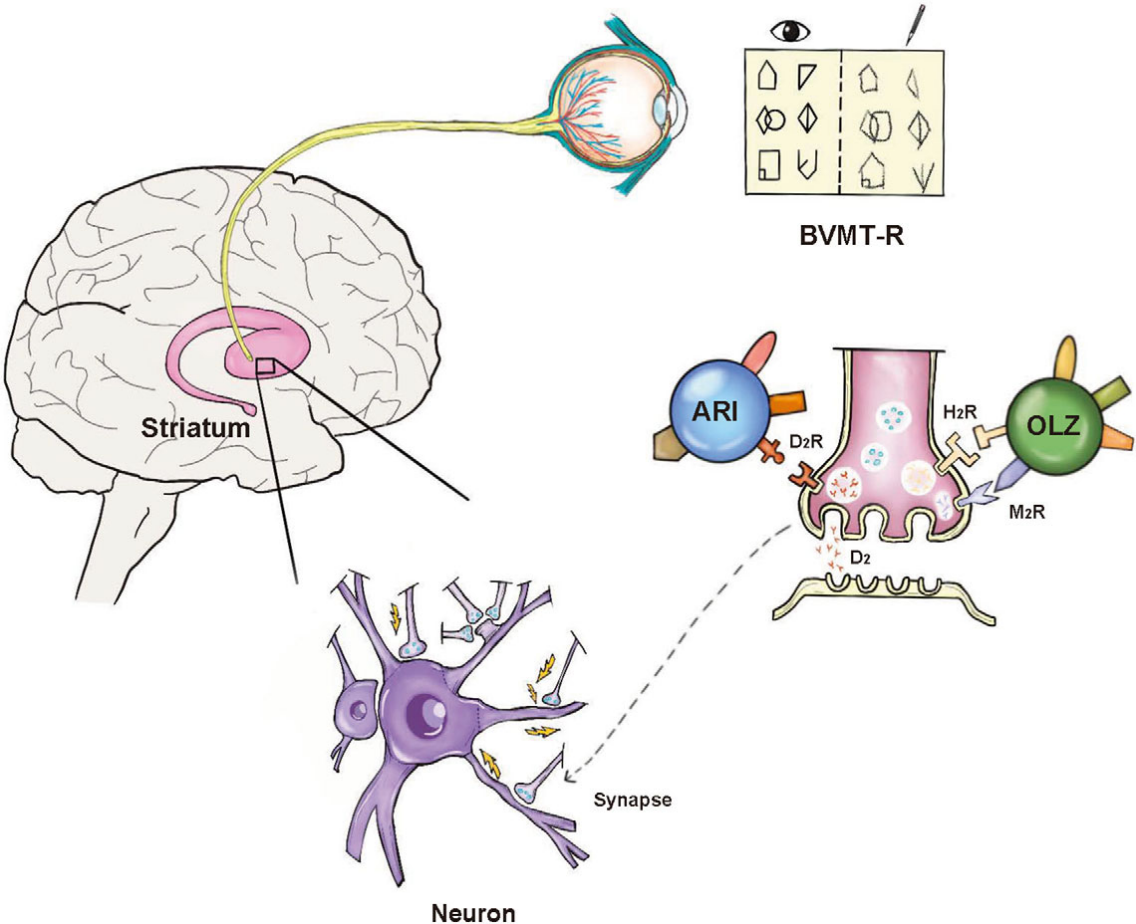
Potential mechanism of aripiprazole in improving visual learning. BVMT-R: Brief Visuospatial Memory Test-Revised. The BVMT-R is a test of visual memory. The subject s shown ten cards that contain abstract geometrical designs. After the presentation of each card, the subject is asked to draw it from memory [57]. ARI: Aripiprazole; OLZ: Olanzapine; D_2_: Dopamine D2; D_2_R: Dopamine D (2)-receptor; H_1_R: Histamine H (1)-receptor; M_3_R: muscarinic M (3)-receptor.

The mechanisms by which aripiprazole affects dopamine synthesis in human brains remain unclear [44]. Animal studies show that repeated aripiprazole administration increases striatal dopamine in young but not adult rats [45], consistent with evidence that antipsychotic mechanisms differ between adolescents and adults [9]. As CHR individuals are generally younger than those with schizophrenia, these age-related antipsychotic effects may apply to CHR populations. Other studies indicate antipsychotics may impact visual function, with evidence that medication can cause visual integration deficits in schizophrenia patients [46] and reduce the strength and duration of visual aftereffects, influencing perceptions and beliefs [47]. Given suggestions that evaluating the visual system could help predict conversion among CHR individuals [48], we hypothesize a psychobiological mechanism underlying the differential cognitive outcomes with aripiprazole versus olanzapine in this population (Figure 2). The negative effects of olanzapine on H1 and M3 receptors that impair cognition may also contribute [49, 50]. We acknowledge this hypothesized mechanism is speculative but aim to broaden the discussion on neurobiological and psychopharmacological mechanisms underlying neurocognition in CHR populations.

### Relationship between neurocognitive changes and changes in functional outcomes

According to this study, the main group effect of AP− is much higher than that of olanzapine. We also demonstrate that CHR individuals have a strong main effect of time on increasing GAF scores. The results of this study confirm previous findings that AP+ individuals do not demonstrate superior functional levels compared to those of AP− CHR individuals and even show a greater risk of poor functional outcomes [10, 16]. Additionally, we discovered evidence that supports previous findings that decline in functioning affects the likelihood of developing psychosis [4, 10].

### Relationship between neurocognitive changes and changes in clinical symptoms

While some studies suggest cognitive impairment is a distinct dimension from psychotic symptoms [34, 51], others demonstrate neurocognitive changes can be associated with clinical manifestations [52, 53]. In this CHR sample, relationships between neurocognition and symptoms/functioning appeared both overlapping and separate. We tend to support a neurodevelopmental hypothesis of schizophrenia [54], given evidence that neurocognitive abnormalities in CHR adolescents are more closely associated with conversion to psychosis than they are in adults [9]. Given that prior studies indicate visual learning (BVMT-R) may mark psychosis risk of CHR individuals [55], our findings that BVMT-R is differentially impacted by aripiprazole versus olanzapine further underscore its importance in this population. Further research should examine links between specific neurocognitive domains like BVMT-R and clinical symptoms in this population.

### Limitations

This study has some limitations. First, as a non-randomized, observational study, it is unclear whether the observed cognitive changes are due to pharmacological treatment, underlying illness severity, or their combined effects. Second, as a typical of prospective cohorts, there was a high attrition rate. We used LMMs to address missing data and examine longitudinal trajectories, as they consider repeated measures and data dependencies. However, given the high attrition, adherence may have decreased in later follow-up. Third, our groups were matched only on age and sex, not on symptom severity. This clinical reality reflects that individuals with more severe symptoms are often more likely to receive antipsychotic treatment, which may influence cognitive trajectories. Fourth, antidepressant use, though brief, may have affected outcomes but sample sizes precluded detailed analysis. Finally, methodological constraints precluded additional validation of cognitive and clinical sequences. Thus, findings could not determine the directionality between neurocognitive and symptom changes.

## Conclusions

CHR individuals showed improvements in symptoms, cognition, and functioning regardless of medication status. However, the most notable gains occurred in unmedicated individuals. Compared to olanzapine, aripiprazole may enhance visual learning. Our findings imply antipsychotics should be used cautiously in CHR to prevent impairments in neurocognition or symptom exacerbation. This supports current guidelines like NICE [56] that do not prioritize antipsychotics as first-line preventive therapeutics for CHR. Further research on neurocognitive factors predisposing to psychosis will be key to enabling early detection and effective prevention in CHR populations.

## Supporting information

10.1192/j.eurpsy.2025.2459.sm001 Zeng et al. supplementary material 1 Zeng et al. supplementary material

10.1192/j.eurpsy.2025.2459.sm002 Zeng et al. supplementary material 2 Zeng et al. supplementary material

## Data Availability

Data will be made available on request.

## Supplementary material

The supplementary material for this article can be found at http://doi.org/10.1192/j.eurpsy.2025.2459.
