# Supplementary material for: The impact of aripiprazole on neurocognitive function in individuals at clinical high risk for psychosis: A comparison with olanzapine and non-antipsychotic treatment
Source: Eur Psychiatry. 2025 May 22;68(1):e69. doi: 10.1192/j.eurpsy.2025.2459 (PMC12188342; doi:10.1192/j.eurpsy.2025.2459)
Supplement: Zeng et al. supplementary material 1 — Zeng et al. supplementary material [file S0924933825024599sup001.docx]

**Supplementary Figure 1. SIPS scores at baseline, 8weeks and 1 year after treatment**


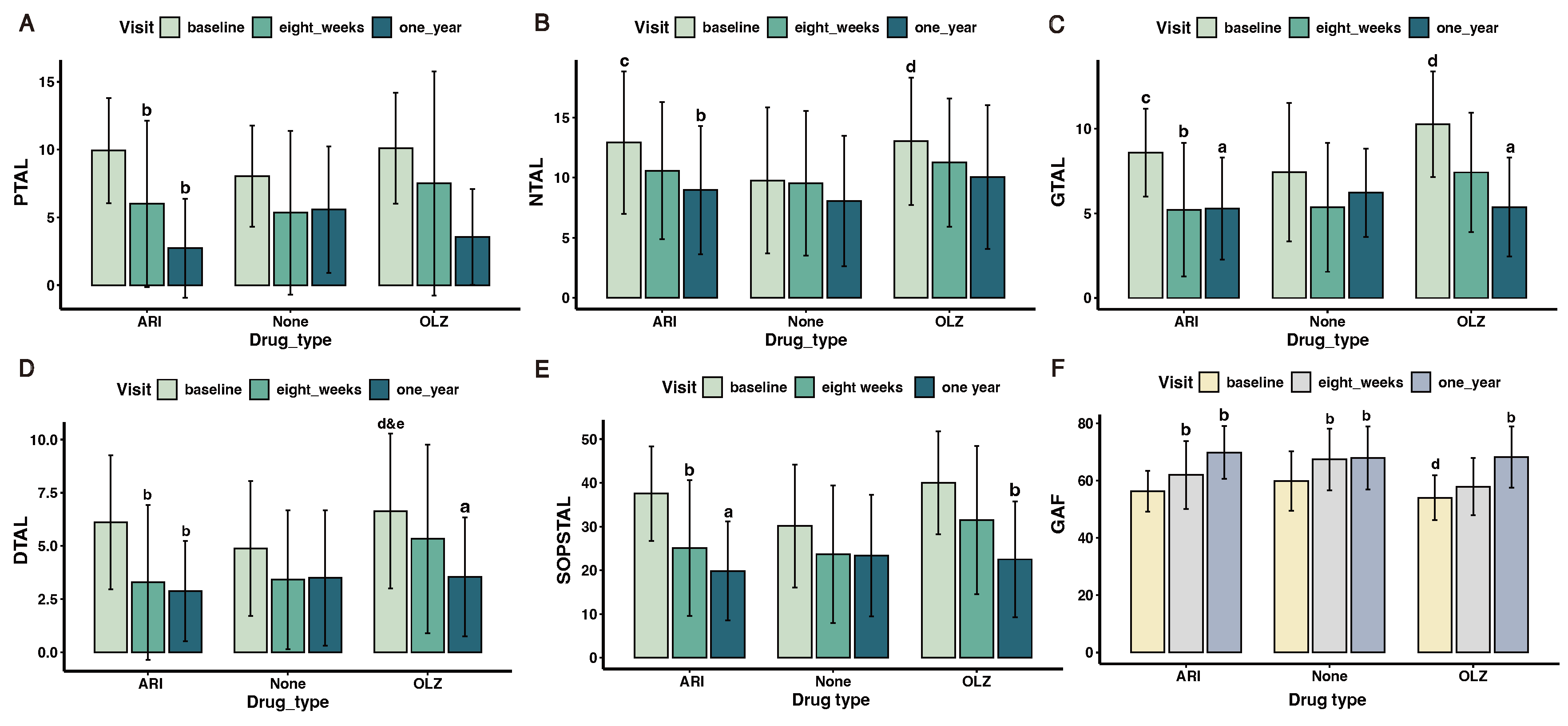


1A. Total score of positive symptoms(PTAL); 1B. Total score of negative symptoms (NTAL); 1C. Total score of disorganization symptoms(DTAL）;1D. Total score of general symptoms(GTAL); 1E. Differences in the total score of SOPS(SOPSTAL); 1F. Differences in the total score of Global Assessment of Functioning(GAF).

Data are expressed as mean±SD; Ari: Aripiprazole group; OLZ: Olanzapine group;

a = Decreased within the group compared to baseline, p＜0.001; b = Decreased within the group compared to baseline, p＜0.05;

Baseline: c = Aripiprazole versus Non-Antipsychotic, p＜0.05; d = Olanzapine versus Non-Antipsychotic, p＜0.05; e = Aripiprazole versus Olanzapine, p＜0.05.

**Supplementary Table 1. Linear Mixed-effects models (LMMs) of SIPS**

| **Domain [β(SE)/F]** | **PTAL** | **NTAL** | **DTAL** | **GTAL** | **SOPSTAL** | **GAF** |
| --- | --- | --- | --- | --- | --- | --- |
| **Ari-Olan** | -0.56(1.01) | -0.64(1.28) | -1.67(0.74)* | -0.53(0.73) | -3.38(2.93) | 2.76(1.99) |
| **None-Olan** | -1.24(1.16) | -2.17(1.46) | -2.86(0.85)*** | -1.76(0.83)* | -8.05(3.34)* | 4.85(2.43)* |
| **8weeks-Baseline** | -2.82(1.41)* | -2.31(1.77) | -2.63(1)** | -1.16(1.03) | -8.89(4.08)* | 4.39(2.77) |
| **1year-Baseline** | -6.57(1.11)*** | -3.02(1.39)* | -4.81(0.78)*** | -3.17(0.82)*** | -17.62(3.22)*** | 14.62(2.16)*** |
| **Ari-Olan×8weeks-**  **Baseline** | -1.09(1.91) | -0.11(2.4) | -0.76(1.36) | -1.58(1.4) | -3.53(5.53) | 1.14(3.77) |
| **None-Olan×8weeks-Baseline** | 0.32(1.86) | 2.46(2.33) | 0.64(1.32) | -0.22(1.36) | 3.15(5.38) | 2.04(3.55) |
| **Ari-Olan×1year-**  **Baseline** | -0.62(1.52) | -0.74(1.91) | 1.6(1.08) | -0.07(1.12) | 0.19(4.41) | -1.19(2.97) |
| **None-Olan×1year-**  **Baseline** | 3.85(1.72)* | 1.16(2.15) | 3.70(1.22)** | 1.69(1.26) | 10.40(4.96)* | -5.31(3.42) |

**Note.** β:estimated effect; SE: Standard Error; F: F value;

PTAL: Total score of positive symptoms; NTAL: Total score of negative symptoms;

DTAL: Total score of disorganization symptoms; GTAL: Total score of general symptoms;

SOPSTAL: Total score of SOPS; GAF: Global Assessment of Functioning.

*indicates p<0.05; **indicates p<0.01; ***indicates p<0.001
