# Supplementary material for: The impact of aripiprazole on neurocognitive function in individuals at clinical high risk for psychosis: A comparison with olanzapine and non-antipsychotic treatment
Source: Eur Psychiatry. 2025 May 22;68(1):e69. doi: 10.1192/j.eurpsy.2025.2459 (PMC12188342; doi:10.1192/j.eurpsy.2025.2459)
Supplement: Zeng et al. supplementary material 2 — Zeng et al. supplementary material [file S0924933825024599sup002.pdf]

Supplementary Figure 1. SIPS scores at baseline, 8weeks and 1 year after treatment

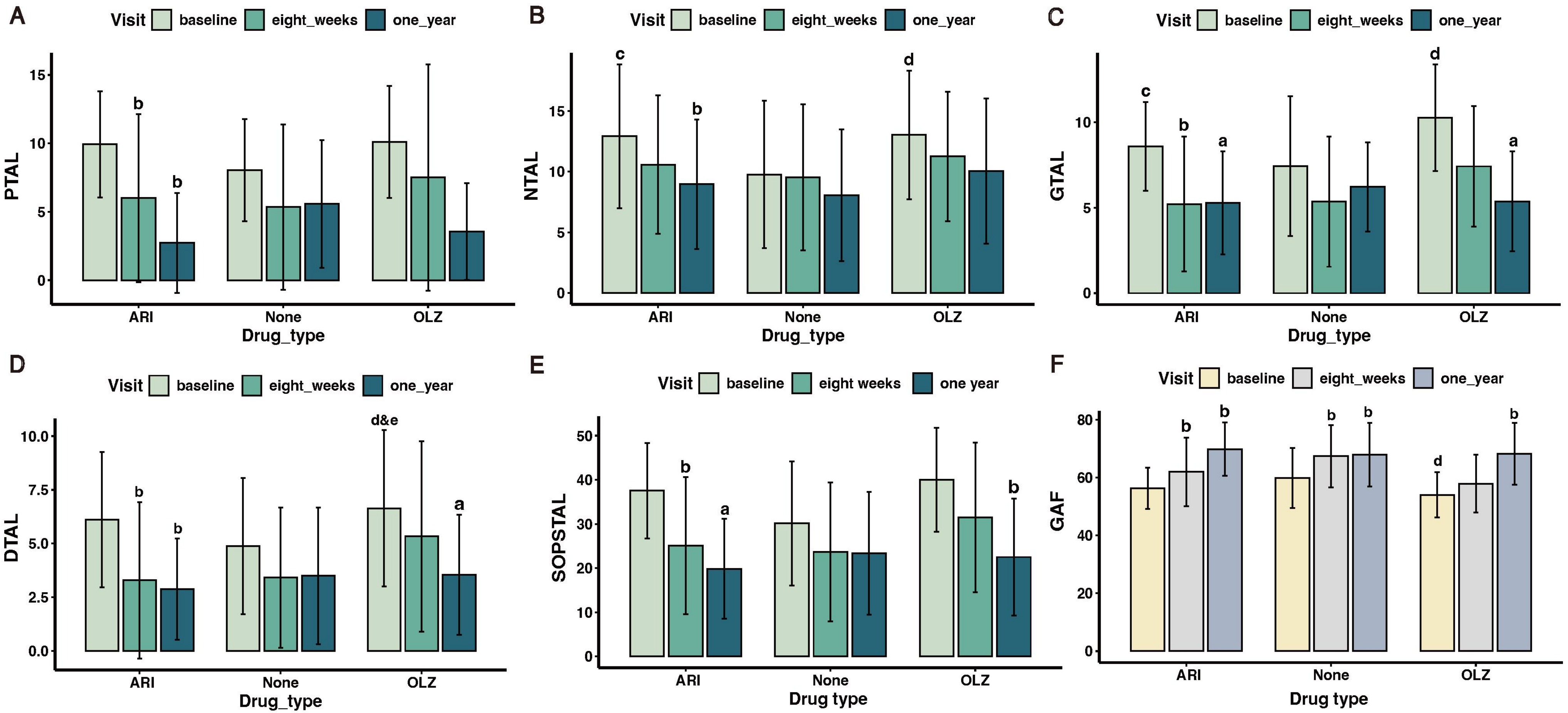

1A. Total score of positive symptoms(PTAL); 1B. Total score of negative symptoms (NTAL); 1C. Total score of disorganization symptoms(DTAL）;1D. Total score of general symptoms(GTAL); 1E. Differences in the total score of SOPS(SOPSTAL); 1F. Differences in the total score of Global Assessment of Functioning(GAF).

Data are expressed as mean±SD; Ari: Aripiprazole group; OLZ: Olanzapine group;

a = Decreased within the group compared to baseline, $p < 0.001$; b = Decreased within the group compared to baseline, $p < 0.05$;

Baseline: c = Aripiprazole versus Non-Antipsychotic, $p < 0.05$; d = Olanzapine versus Non-Antipsychotic, $p < 0.05$; e = Aripiprazole versus Olanzapine, $p < 0.05$.

Supplementary Table 1. Linear Mixed-effects models (LMMs) of SIPS

Domain [β(SE)/F]	PTAL	NTAL	DTAL	GTAL	SOPSTAL	GAF
**Ari-Olan**	-0.56(1.01)	-0.64(1.28)	-1.67(0.74)*	-0.53(0.73)	-3.38(2.93)	2.76(1.99)
**None-Olan**	-1.24(1.16)	-2.17(1.46)	-2.86(0.85)***	-1.76(0.83)*	-8.05(3.34)*	4.85(2.43)*
**8weeks-Baseline**	-2.82(1.41)*	-2.31(1.77)	-2.63(1)**	-1.16(1.03)	-8.89(4.08)*	4.39(2.77)
**1year-Baseline**	-6.57(1.11)***	-3.02(1.39)*	-4.81(0.78)***	-3.17(0.82)***	-17.62(3.22)***	14.62(2.16)***
**Ari-Olan×8weeks-Baseline**	-1.09(1.91)	-0.11(2.4)	-0.76(1.36)	-1.58(1.4)	-3.53(5.53)	1.14(3.77)
**None-Olan×8weeks-Baseline**	0.32(1.86)	2.46(2.33)	0.64(1.32)	-0.22(1.36)	3.15(5.38)	2.04(3.55)
**Ari-Olan×1year-Baseline**	-0.62(1.52)	-0.74(1.91)	1.6(1.08)	-0.07(1.12)	0.19(4.41)	-1.19(2.97)
**None-Olan×1year-Baseline**	3.85(1.72)*	1.16(2.15)	3.70(1.22)**	1.69(1.26)	10.40(4.96)*	-5.31(3.42)

Note. β:estimated effect; SE: Standard Error; F: F value;
PTAL: Total score of positive symptoms; NTAL: Total score of negative symptoms;
DTAL: Total score of disorganization symptoms; GTAL: Total score of general symptoms;
SOPSTAL: Total score of SOPS; GAF: Global Assessment of Functioning.
*indicates $p<0.05$; **indicates $p<0.01$; ***indicates $p<0.001$
